# Understanding Outcomes in Hemodialysis Patients Who Underwent Transcatheter Aortic Valve Replacement With the Latest Devices

**DOI:** 10.1016/j.jacasi.2026.04.032

**Published:** 2026-06-17

**Authors:** Juri Iwata, Masanori Yamamoto, Kei Kamata, Ryo Arita, Tomonari Moriizumi, Toshinobu Ryuzaki, Hikaru Tsuruta, Takahiro Tokuda, Tetsuro Shimura, Shinichi Shirai, Kenichi Ishizu, Yusuke Watanabe, Toru Naganuma, Futoshi Yamanaka, Masahiko Noguchi, Yohei Ohno, Masaki Izumo, Hidetaka Nishina, Masahiko Asami, Gaku Nakazawa, Fumiaki Yashima, Hirofumi Hioki, Yasushi Fuku, Hiroto Suzuyama, Kazumasa Yamasaki, Kenji Nishioka, Daisuke Hachinohe, Toshiaki Otsuka, Norio Tada, Hideyuki Shimizu, Masaki Ieda, Kentaro Hayashida, OCEAN-TAVI Investigators

**Affiliations:** a Department of Cardiology, Keio University School of Medicine, Tokyo, Japan; b Department of Cardiology, Toyohashi Heart Center, Toyohashi, Japan; c Department of Cardiology, Nagoya Heart Center, Nagoya, Japan; d Department of Cardiology, Gifu Heart Center, Gifu, Japan; e Department of Cardiology, Kokura Memorial Hospital, Kokura, Japan; f Department of Cardiology, Teikyo University, Tokyo, Japan; g Department of Cardiology, New Tokyo Hospital, Chiba, Japan; h Department of Cardiology, Shonan Kamakura General Hospital, Kanagawa, Japan; i Department of Cardiology, Tokyo Bay Urayasu Ichikawa Medical Center, Urayasu, Japan; j Department of Cardiology, Tokai University, Isehara, Japan; k Department of Cardiology, St Marianna University School of Medicine, Kawasaki, Japan; l Department of Cardiology, Tsukuba Medical Center Hospital, Tsukuba, Japan; m Division of Cardiology, Mitsui Memorial Hospital, Tokyo, Japan; n Department of Cardiology, Kinki University, Osaka, Japan; o Department of Cardiology, Saiseikai Utsunomiya Hospital, Utsunomiya, Japan; p Department of Cardiology, IMS Tokyo Katsushika General Hospital, Tokyo, Japan; q Department of Cardiovascular Medicine, Kurashiki Central Hospital, Kurashiki, Japan; r Department of Cardiology, Saiseikai Kumamoto Hospital, Kumamoto, Japan; s Department of Cardiology, Sapporo Higashi Tokushukai Hospital, Hokkaido, Japan; t Department of Cardiology, Hiroshima City Hiroshima Citizens Hospital, Hiroshima, Japan; u Department of Cardiology, Sapporo Heart Center, Sapporo Cardiovascular Clinic, Sapporo, Japan; v Department of Hygiene and Public Health, Nippon Medical School, Tokyo, Japan; w Department of Cardiology, Sendai Kousei Hospital, Sendai, Japan; x Department of Cardiovascular Surgery, Keio University School of Medicine, Tokyo, Japan

**Keywords:** aortic valve stenosis, balloon-expandable valves, hemodialysis, self-expandable valves, transcatheter aortic valve replacement

## Abstract

**Background:**

The poor prognosis of hemodialysis (HD) patients following transcatheter aortic valve replacement (TAVR) has been established; however, data on the outcomes in the latest generation of devices remain inconsistent.

**Objectives:**

The authors aimed to compare the 1-year clinical outcomes post-TAVR using the latest generation of devices in HD and non-HD patients.

**Methods:**

From the multicenter registry, 760 HD and 3,928 non-HD patients were identified from the OCEAN-TAVI (Optimized transCathEter vAlvular iNtervention-Transcatheter Aortic Valve Implantation; UMINID:000020423) registry. To minimize differences in baseline characteristics, 1:1 propensity score matching (PSM) was performed (490 patients each). The primary clinical endpoint was all-cause mortality at 1 year. Secondary endpoints included cardiovascular death, stroke, and heart failure rehospitalization.

**Results:**

In the overall cohort, during 208 (41-373) days of follow-up, HD patients had higher 1-year mortality than non-HD patients (105 of 760 [13.8%] vs 189 of 3,928 [4.8%], HR: 2.62; 95% CI: 2.13-3.23; *P* < 0.001); this difference was attenuated (59 of 490 [12.0%] vs 65 of 490 [13.3%], HR: 1.03; 95% CI: 0.75-1.42; *P* = 0.858) following well-balanced PSM. There were no significant differences in any secondary endpoints between the 2 groups after PSM; however, HD remained an independent predictor of 1-year mortality in a multivariate analysis of the cohort before PSM.

**Conclusions:**

The poor prognostic value of HD was attenuated after adjusting for baseline risk factors. These findings suggest that the poor outcomes of HD patients result from the burden of multiple comorbidities in addition to the HD risk itself. Considering TAVR as a treatment option for exceptionally high-risk populations will aid in the careful patient selection and realistic prognostic assessments.

Aortic stenosis is highly prevalent among patients undergoing hemodialysis (HD). In these patients, advanced comorbidities and frailty are major contributors to poor outcomes following surgical aortic valve replacement (SAVR).^1^, ^2^, ^3^, ^4^ Transcatheter aortic valve replacement (TAVR) is a less invasive alternative for patients with a wide range of surgical risks, providing survival benefits and procedural safety to many populations.^5^, ^6^, ^7^, ^8^ However, patients on HD frequently present with extensive vascular calcification, frailty, and multiple comorbidities, which make TAVR technically and clinically complex. Indeed, previous studies have reported higher early- and mid-term mortality and bleeding complications in TAVR than in non-HD patients.^9^, ^10^, ^11^ Despite these characteristics, most previous reports have been based on early-generation devices, and whether contemporary device improvements have narrowed the outcome gap between HD and non-HD patients remains unclear. Whether the poor outcomes of patients on HD are driven primarily by the uremic condition itself, the burden of multiple comorbidities, or both remains undetermined. In this context, evaluating outcomes with the latest-generation devices will provide novel insights into prognostic determinants in HD patients. Therefore, this study aimed to compare short-term outcomes and 1-year prognosis after TAVR with the latest-generation devices between HD and non-HD patients from a nationwide multicenter registry.

## Methods

### Study cohort

The OCEAN-TAVI (Optimized transCathEter vAlvular iNtervention-Transcatheter Aortic Valve Implantation) registry is an ongoing nationwide prospective multicenter cohort study involving patients with aortic stenosis who underwent TAVR at 22 collaborating centers across Japan. The present analysis retrospectively included patients who underwent TAVR with the latest valves, SAIEN 3 Ultra RESILIA (Edwards Lifesciences) or Evolut FX (Medtronic), between May 2022 and December 2024 at participating institutes. Patients who underwent TAVR with Navitor (Abbott), which is not reimbursed for HD patients in Japan, or TAVR for degenerated surgical bioprostheses or transcatheter heart valves were excluded. The participating centers were mandated to enroll patients in the registry, adhere to the registry protocol, and provide patient information and clinical outcomes during follow-up. The study protocol was approved by the ethics committee of each center, and all patients provided written informed consent for participation. This study complied with the principles of the Declaration of Helsinki. The study was registered with the University Hospital Medical Information Network Clinical Trial Registry and accepted by the International Committee of Medical Journals (UMINID:000020423).

### Data collection and endpoint definitions

Patients on HD were included in both maintenance and peritoneal groups. Baseline clinical, procedural, and follow-up data were prospectively recorded in a web-based database at each participating center. Regular follow-ups were scheduled before the procedure, at discharge, and 1 year after TAVR. Comprehensive clinical follow-up data were collected from medical records at each center, referring physician documentation, and/or telephone interviews. The web-based database was systematically audited by the data committee members to ensure completeness and accuracy, and to maintain data integrity. In cases of incomplete or inaccurate data, all participating centers were required to respond to queries. The follow-up period was defined by setting the date of TAVR as day 0 and the date of the last survival confirmation as the end of the follow-up period.

### Endpoints for survival analysis

Clinical endpoints included all-cause mortality, cardiovascular mortality, heart failure rehospitalization (HFH), and stroke. If follow-up was terminated before these endpoints were reached, they were censored.

Transthoracic echocardiography was performed before, at discharge, and 1 year after TAVR. Following the updated Valve Academic Research Consortium-3 criteria,^12^ prosthesis-patient mismatch (PPM) was assessed as the prosthesis effective orifice area indexed to the body surface area and categorized as severe (≤0.65 cm^2^/m^2^ in the population without obesity [body mass index <30 kg/m^2^]; ≤0.55 cm^2^/m^2^ in the population with obesity [body mass index ≥30 kg/m^2^]) or moderate (>0.65 to 0.85 cm^2^/m^2^ in the population without obesity; >0.55 to 0.70 cm^2^/m^2^ in the population with obesity). All procedural complications were defined according to the Valve Academic Research Consortium-3 criteria,^12^ and relevant data were collected systematically.

### Statistical analysis

Categorical variables were reported as frequencies and percentages, and differences were evaluated using the chi-square test or 2-tailed Fisher’s exact test. Continuous variables were presented as mean ± SD or median (IQR), with between-group comparisons performed using the 2-sample Student's *t*-test or Mann-Whitney *U* test, as appropriate. Time-to-event curves were generated using the Kaplan-Meier method (censored at death or last valid contact in patients awaiting the subsequent follow-up or consent withdrawal). A Cox proportional hazard model was used to calculate HRs and 95% CIs for all-cause mortality without frailty or mix model because the analysis is intended to examine the fixed factor of dialysis status (rather than renal function itself), and the analysis was conducted with facility names blinded. If the mortality rates in both groups (dialysis and nondialysis) increase visually at the same rate over time following TAVR, we consider the proportional hazards hold. The mechanism of missingness was assumed to be missing completely at random, whereby the probability of missingness is independent of both observed and unobserved data. The assumption of missing completely at random was assessed using Little’s Missing Completely at Random test. There was no evidence against the Missing Completely at Random assumption (Little’s test, *P* = 0.32). SPSS version 29 (SPSS Inc) was used for all the statistical analyses.

We used propensity score matching (PSM) to control for confounding factors arising from baseline differences between HD and non-HD patients undergoing TAVR. The propensity score was calculated using a multivariate logistic regression model based on the following 24 variables that could have affected study outcomes: age; body mass index (kg/m^2^); Society of Thoracic Surgeons (STS) Predicted Risk of Mortality (%); clinical frailty scale (CFS) ≥4; NYHA functional class III or IV; hypertension; diabetes mellitus; peripheral artery disease; chronic obstructive pulmonary disease; atrial fibrillation; hemoglobin level (g/dL); history of coronary artery disease; history of stroke; previous pacemaker implantation; echocardiographic variables such as aortic valve area (m^2^), aortic valve mean pressure gradient (PG) (mm Hg), left ventricular ejection fraction (%), moderate or severe aortic regurgitation, moderate or severe mitral regurgitation, and moderate or severe tricuspid regurgitation; computed tomography variables, such as annulus area (mm^2^); and procedural variables such as access site (transfemoral approach or non), emergency (elective or non), and valve type (SAPIEN 3 Ultra RESILIA or Evolut FX). Herein, a 1:1 greedy nearest-neighbor matching protocol with a caliper of 0.2 was used. Absolute standardized differences were estimated for all baseline variables, and values <0.10 were considered an indicator of good balance—results for the overall matched cohort and for cohorts by annulus size. In the overall cohort, before PSM, we fit multivariate logistic regression models.

In the overall cohort, before PSM, we fit multivariate logistic regression models. The sample size consisted of 760 HD participants and 3,928 non-HD participants.

Subgroup analyses were performed to assess the consistency of the treatment effect across clinically relevant subgroups, including annulus area (<430 vs ≥430 mm^2^), sex, valve type (Evolut FX vs S3UR), mean PG (<20 vs ≥20 mm Hg), PPM (none or moderate vs severe), age (<75 vs ≥75 years), body surface area (<1.5 vs ≥1.5 m^2^), CFS (<4 vs ≥4), and STS score (<4%, 4%</≥8%, ≥8%). Treatment effects within each subgroup were estimated using Cox proportional hazards models, and HRs with 95% CIs were reported. Heterogeneity of treatment effects was evaluated by including interaction terms between treatment assignment and subgroup variables in the model. All subgroup analyses were considered exploratory, and no adjustments were made for multiple comparisons.

## Results

### Study population and baseline characteristics

**Figure 1:**
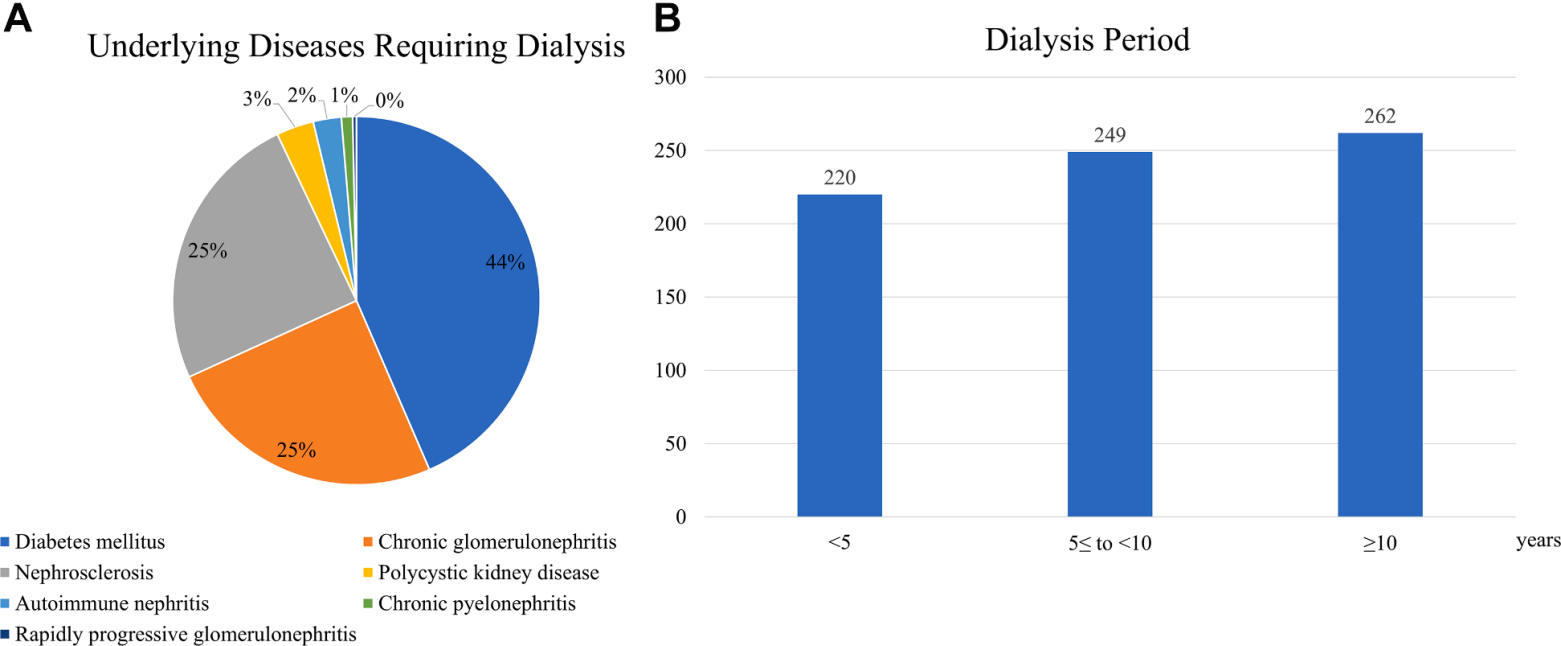
Baseline Characteristics of HD Patients in the OCEAN-TAVI Registry This figure summarizes background factors such as the reasons for starting hemodialysis (HD) and the duration of HD among patients with HD. (A) Causes of HD. (B) Duration of HD. OCEAN-TAVI = Optimized transCathEter vAlvular iNtervention-Transcatheter Aortic Valve Implantation.

**Figure 2:**
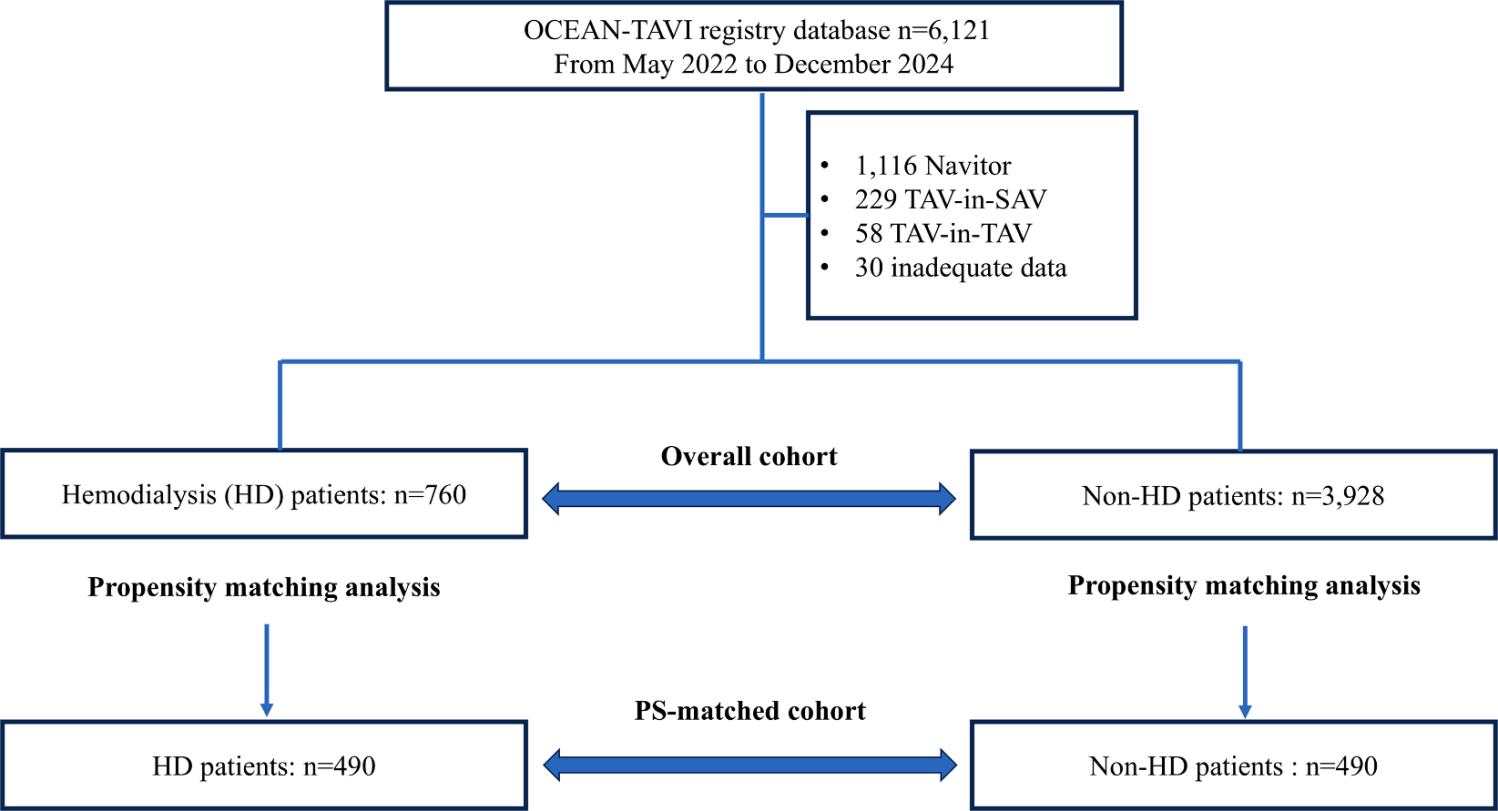
Study Flow Diagram From the OCEAN-TAVI Registry This figure outlines patient selection from the OCEAN-TAVI registry between May 2022 and December 2024. Among 6,121 patients undergoing TAVR, those with incomplete data, TAV-in-SAV, and TAV-in-TAV procedures were excluded. The final cohort included 760 HD and 3,928 non-HD patients. After propensity score matching, 490 matched pairs were analyzed to compare clinical outcomes between HD and non-HD groups. OCEAN-TAVI = Optimized transCathEter vAlvular iNtervention-Transcatheter Aortic Valve Implantation; PS = propensity score; SAV = surgical aortic valve; TAV = transcatheter aortic valve; TAVR = transcatheter aortic valve replacement; other abbreviation as in Figure 1.

Among 6,121 patients who underwent TAVR, 4,688 met the inclusion criteria. There were 760 HD patients (16.2%). The factors and duration of dialysis are summarized in Figure 1. The median dialysis duration was 7 (3-13) years. The most common underlying disease leading to dialysis was diabetes, accounting for 334 of 760 (44%), followed by chronic glomerulonephritis, nephrosclerosis, polycystic kidney disease, autoimmune nephritis, chronic pyelonephritis, and rapidly progressive glomerulonephritis. One-to-one PSM resulted in 490 patients in the HD and non-HD groups (Figure 2). The baseline characteristics of the unmatched and matched cohorts are shown in Table 1. Before PSM, the groups differed in numerous parameters, including CFS and other comorbidities. The HD group was younger (80.0 ± 6.2 vs 84.8 ± 5.6 years, *P* < 0.001), higher proportion of men (477 of 760 [62.8%] vs 1,934 of 3,928 [35.5%], *P* < 0.001), smaller body mass index (21.2 ± 3.9 kg/m^2^ vs 22.4 ± 3.8 kg/m^2^, *P* < 0.001), higher STS score (11.6% [7.5%-17.4%] vs 6.2% [4.1%-9.8%], *P* < 0.001) than the non-HD group.

**Table 1:** Baseline Characteristics of the Before-Matching and Matched Cohorts

	Before-Matched Cohort			Matched Cohort			ASD
	HD (n = 760)	Non-HD (n = 3,928)	*P* Value	HD (n = 490)	Non-HD (n = 490)	*P* Value	
Age, y	80.0 ± 6.2	84.8 ± 5.6	<0.001	81.2 ± 5.1	81.5 ± 7.2	0.503	0.047
Male	477 (62.8)	1394 (35.5)	<0.001	291 (59.4)	296 (60.4)	0.745	0.021
Body mass index, kg/m^2^	21.2 ± 3.3	22.4 ± 3.8	<0.001	21.4 ± 3.2	21.5 ± 3.5	0.431	0.033
STS-PROM score, %	11.6 [7.5-17.4]	6.2 [4.1-9.8]	<0.001	10.4 [6.9-16.0]	7.6 [4.2-15.1]	<0.001	0.050
Clinical frailty scale ≥4	449 (59.1)	1971 (50.2)	<0.001	275 (56.1)	275 (56.1)	0.93	0.024
NYHA functional class III or IV	251 (33.0)	1050 (26.7)	<0.001	162 (33.1)	155 (31.6)	0.633	0.031
Hemoglobin, g/dL	11.2 [10.3-12.1]	11.7 [10.5-12.9]	<0.001	11.2 [10.3-12.2]	11.2 [9.8-12.4]	0.27	0.055
Brain natrium peptide, pg/ml	782 [340-1698]	162 [73-393]	<0.001	701 [318-1492]	281 [117-622]	<0.001	0.144
Concomitant diseases							
Hypertension	589 (77.5)	3085 (78.5)	0.524	375 (76.5)	389 (79.4)	0.281	0.069
Diabetes mellitus	314 (41.3)	1140 (29.0)	<0.001	194 (39.6)	199 (40.6)	0.745	0.021
Dyslipidemia	413 (53.3)	390 (50.4)	0.484	229 (46.7)	263 (53.7)	0.03	
Chronic obstructive pulmonary disease	60 (7.9)	289 (7.4)	0.605	31 (6.3)	46 (9.4)	0.075	0.114
Atrial fibrillation	178 (23.4)	913 (23.2)	0.916	115 (23.5)	132 (26.9)	0.211	0.08
Coronary artery disease	337 (44.3)	1107 (28.2)	<0.001	192 (39.2)	191 (39.0)	0.948	0.004
Peripheral artery disease	252 (33.2)	419 (10.7)	<0.001	123 (25.1)	114 (23.3)	0.502	0.043
Carotid stenosis	47 (6.1)	40 (5.2)	0.050	41 (8.4)	48 (9.8)	0.620	
Previous history							
Smoking	306 (40.5)	940 (24.1)	<0.001	191 (39.0)	217 (44.3)	0.024	
History of PCI	199 (26.2)	617 (15.7)	<0.001	100 (20.4)	119 (24.3)	0.145	
History of CABG	67 (8.8)	75 (1.9)	<0.001	33 (6.7)	26 (5.3)	0.347	
History of myocardial infarction	61 (8.0)	168 (4.3)	<0.001	24 (4.9)	48 (9.8)	0.003	
History of stroke	115 (15.1)	414 (10.5)	<0.001	85 (11.0)	90 (11.6)	0.561	0.050
History of bleeding	52 (6.8)	159 (4.1)	<0.001	26 (5.3)	36 (7.3)	0.253	
Previous pacemaker	49 (6.4)	165 (4.2)	0.007	26 (5.3)	24 (4.9)	0.772	0.019
Echocardiographic data							
Aortic valve area, cm^2^	0.69 ± 0.19	0.67 ± 0.19	<0.001	0.69 ± 0.18	0.68 ± 0.20	0.141	0.058
Index aortic valve area, cm^2^/m^2^	0.46 ± 0.12	0.46 ± 0.13	0.170	0.46 ± 0.11	0.45 ± 0.13	0.175	
Peak flow velocity, m/s	4.1 ± 0.65	4.4 ± 0.71	<0.001	4.2 ± 0.63	4.2 ± 0.71	0.634	
Aortic valve peak gradient, mm Hg	70.3 ± 21.8	78.5 ± 25.6	<0.001	72.0 ± 21.3	72.9 ± 23.5	0.634	
Aortic valve mean gradient, mm Hg	40.3 ± 13.6	45.7 ± 16.5	<0.001	41.1 ± 13.4	42.3 ± 14.7	0.145	0.084
Left ventricular ejection fraction, %	53.8 ± 13.8	60.6 ± 11.6	<0.001	55.2 ± 13.0	55.2 ± 14.5	0.457	0.003
Aortic regurgitation ≥ moderate	94 (12.4)	412 (10.5)	0.302	57 (11.6)	69 (14.1)	0.252	0.073
Mitral regurgitation ≥ moderate	150 (19.7)	487 (12.4)	<0.001	86 (17.6)	87 (17.8)	0.996	0
Tricuspid regurgitation ≥ moderate	93 (12.2)	390 (9.9)	0.029	57 (11.6)	67 (13.7)	0.387	0.063
Computed tomography							
Annulus area	440 [382-495]	402 [356-458]	<0.001	436 [382-487]	433 [374-493]	0.695	0.026
Area ≤430 mm^2^	339 (44.6)	2475 (63.0)	<0.001	229 (46.7)	235 (48.0)	0.701	
Perimeter, mm	76.1 [71.0-80.7]	72.8 [68.6-77.7]	<0.001	75.7 [70.9-80.2]	75.2 [70.1-81.0]	0.638	

Values are mean ± SD, or n (%). *P* values from Fisher's test (2 × 2 comparison), chi-square test (n × 2 comparisons), or Student's *t*-tests (continuous parameters). The numbers in brackets indicate the percentage of the total.

ASD = absolute standardized difference; CABG = coronary artery bypass grafting; HD = hemodialysis; PCI = percutaneous coronary intervention; STS-PROM = Society of Thoracic Surgeons Predicted Risk of Mortality.

Moreover, the HD group was frail, with a higher proportion of NYHA functional class III or IV, lower hemoglobin levels, and higher BNP levels. Concomitant and previous histories significantly differed; HD patients had diabetes mellitus, coronary artery disease, peripheral artery disease, a history of smoking, percutaneous coronary intervention, coronary artery bypass grafting, myocardial infarction, stroke, bleeding (all *P* < 0.001), or pacemaker implantation (*P* = 0.007). In echocardiographic assessments, patients in the HD group exhibited a larger aortic valve area (0.69 ± 0.19 vs 0.67 ± 0.19 cm^2^, *P* < 0.001), lower peak flow velocity (4.1 ± 0.65 vs 4.4 ± 0.71 m/s, *P* < 0.001), lower mean PG (40.3 ± 13.6 vs 45.7 ± 16.5 mm Hg, *P* < 0.001), and lower left ventricular ejection fraction (53.8% ± 13.8% vs 60.6% ± 11.6%, *P* < 0.001) compared with non-HD patients. In computed tomography analysis, HD patients showed a larger annulus area (440 [382-495] vs 402 [356-458] mm^2^, *P* < 0.001) and a longer perimeter (76.1 [71.0-80.7] vs 72.8 [68.6-77.7] mm, *P* < 0.001). After PSM, the patient characteristics of the HD and non-HD groups were well balanced, with absolute standardized differences <0.10 for most baseline characteristics, except for comorbid chronic obstructive pulmonary disease (0.114).

### Procedural characteristics, complications, and echocardiographic outcomes

The procedures, complications, and echocardiographic outcomes of the prematched and matched cohorts are shown in Table 2. Before the PSM cohort, the HD group had a lower rate of the transfemoral approach (681 of 760 [89.6%] vs 3,743 of 3,928 [95.3%], *P* < 0.001), the puncture approach (643 of 760 [84.5%] vs 3,660 of 3,928 [93.2%], *P* < 0.001), and local anesthesia (530 of 760 [69.7%] vs 2,896 of 3,928 [73.7%], *P* = 0.023). In addition, the HD group that used Evolut FX less frequently (222 of 760 [29.2%] vs 1,475 of 3,928 [37.6%], *P* < 0.001) had a larger valve size and less frequent post dilatation. These differences were well balanced following PSM.

**Table 2:** Procedure, Complications, and Echocardiographic Outcomes

	Before-Matched Cohort			Matched Cohort			ASD
	HD (n = 760)	Non-HD (n = 3,928)	*P* Value	HD (n = 490)	Non-HD (n = 490)	*P* Value	
Transfemoral	681 (89.6)	3,743 (95.3)	<0.001	452 (92.2)	438 (89.4)	0.121	0.018
Nonelective	52 (6.8)	300 (7.6)	0.446	38 (7.8)	53 (10.8)	0.099	0.105
Elective	708 (93.2)	3,628 (92.4)	0.597	452 (92.2)	437 (89.2)	0.081	
Urgent	41 (5.4)	254 (6.5)		33 (6.7)	39 (8.0)		
Emergent	11 (1.4)	45 (1.1)		5 (1.0)	14 (2.9)		
Salvage	0 (0)	1 (0)		0 (0)	0 (0)		
Valve type							0.018
SAPIEN 3 Ultra RESILIA	538 (70.8)	2,453 (62.4)	<0.001	345 (70.4)	341 (69.6)	0.780	
Evolut FX	222 (29.2)	1475 (37.6)		145 (29.6)	149 (30.4)		
Valve size, mm							
20	29 (3.8)	257 (6.5)	<0.001	23 (4.7)	25 (5.1)	0.564	
23	217 (28.6)	1507 (38.4)		146 (29.8)	159 (32.4)		
26	332 (43.7)	1,446 (36.8)		214 (43.7)	194 (39.6)		
29	162 (21.3)	632 (16.1)		92 (19.8)	90 (18.4)		
34	20 (2.6)	85 (2.2)		15 (3.1)	22 (4.5)		
Local anesthesia	530 (69.7)	2,896 (73.7)	0.023	335 (68.4)	330 (67.3)	0.732	
Puncture	643 (84.6)	3,660 (93.2)	<0.001	428 (87.3)	418 (85.3)	0.631	
Predilatation	141 (15.9)	196 (13.3)	0.077	173 (35.3)	169 (34.5)	0.789	
Post dilatation	101 (11.4)	260 (17.6)	<0.001	77 (15.7)	75 (15.3)	0.860	
Echocardiographic data							
Effective orifice area, cm^2^	1.92 ± 0.51	1.89 ± 0.52	0.030	1.88 ± 0.49	1.88 ± 0.51	0.874	
Index effective orifice area, cm^2^/m^2^	1.28 ± 0.34	1.29 ± 0.36	0.858	1.27 ± 0.33	1.26 ± 0.34	0.783	
Peak flow velocity, m/s	2.0 ± 0.42	2.0 ± 0.45	0.231	2.0 ± 0.42	1.9 ± 0.45	0.007	
Mean pressure gradient, mm Hg	8.2 ± 3.7	8.6 ± 4.3	0.097	8.5 ± 3.8	8.1 ± 4.0	0.037	
Mean pressure gradient ≥20 mmHg	12 (1.6)	76 (2.0)	0.506	9 (1.9)	8 (1.7)	0.820	
Prosthesis-patient mismatch							
None	689 (93.0)	3,558 (92.9)	0.858	448 (93.5)	423 (89.8)	0.116	
Moderate	46 (6.2)	231 (6.0)		27 (5.6)	42 (8.9)		
Severe	6 (0.8)	39 (1.0)		4 (0.8)	6 (1.3)		
Paravalvular leakage ≥ mild	162 (21.5)	872 (22.5)	0.554	111 (22.7)	104 (21.2)	0.340	
Paravalvular leakage							
None	295 (39.2)	1,707 (44.1)	0.004	190 (38.8)	226 (46.1)	0.004	
Trivial	295 (39.2)	1,292 (33.4)		185 (37.8)	151 (30.8)		
Mild	155 (20.6)	784 (20.3)		107 (21.8)	90 (18.4)		
Moderate	7 (0.9)	86 (2.2)		4 (0.8)	14 (2.9)		
Severe	0 (0)	2 (0.1)		0 (0)	0 (0)		
Ent days	6 [4-10]	6 [4-9]	0.018	6 [4-10]	7 [5-15]	<0.001	
In-hospital death	26 (3.4)	44 (1.1)	<0.001	12 (2.4)	17 (3.5)	0.346	
Cardiovascular death	8 (1.1)	25 (0.6)	<0.001	3 (0.6)	8 (1.6)	0.317	
Open surgery	0 (0)	1 (0.1)	0.122	0 (0)	3 (0.6)	0.083	
Life-threatening and major bleeding	62 (8.2)	163 (4.1)	<0.001	38 (7.8)	35 (7.1)	0.715	
Disabling stroke	12 (1.6)	365 (0.9)	0.097	8 (1.6)	8 (1.6)	1.00	
Major vascular complication	18 (2.4)	86 (2.2)	0.759	7 (1.4)	26 (5.3)	<0.001	
Tamponade	3 (0.4)	17 (0.4)	0.883	1 (0.2)	8 (1.6)	0.019	
Valve embolization	1 (0.1)	4 (0.1)	0.818	0 (0)	0 (0)	NA	
New pacemaker implantation	59 (7.8)	233 (5.9)	0.056	39 (8.0)	27 (5.5)	0.126	
Discharge NYHA functional class III or IV	17 (2.0)	52 (4.0)	0.011	11 (2.2)	16 (3.3)	0.599	

Values are mean ± SD, or n (%). *P* values from Fisher's test (n × 2 comparison), chi-square test (n × 2 comparisons), or Student's *t*-tests (continuous parameters). *P* values were not calculated because no events occurred in either group. The numbers in brackets indicate the percentage of the total.

NA = not applicable. Other abbreviations as in Table 1.

Regarding in-hospital clinical outcomes, among the overall cohort, all-cause death, cardiovascular death, and life-threatening/major bleeding were significantly higher in the HD than non-HD groups (26 of 760 [3.4%] vs 44 of 3,928 [1.1%], *P* < 0.001; 8 of 760 [1.1%] vs 25 of 3,928 [0.6%], *P* < 0.001; 62 of 760 [8.2%] vs 163 of 3,928 [4.1%], *P* < 0.001, respectively). These differences were eliminated by PSM. Instead, tamponade and major vascular complications were more frequently observed in the non-HD group.

After the PSM cohort, postprocedural echocardiography revealed higher peak flow velocity and mean PG (2.0 ± 0.42 m/s vs 1.9 ± 0.45 m/s; *P* = 0.007; 8.5 ± 3.8 vs 8.1 ± 4.0 mm Hg; *P* = 0.037). The HD group had a higher incidence of paravalvular leakage (PVL) (none, 190 of 490 [38.8%] vs 226 of 490 [46.1%]; trivial, 185 of 490 [37.8%] vs 151 of 490 [30.8%]; mild, 107 of 490 [21.8%] vs 90 of 490 [18.4%]; moderate, 4 of 490 [0.8%] vs 14 of 490 [2.9%]; severe, 0 of 490 [0%] vs 0 of 490 [0%], *P* = 0.004) than the non-HD group. However, there were no significant differences in PVL ≥ mild, mean PG ≥20 mm Hg, or the incidence of PPM between the groups.

Echocardiographic and other clinical data 1 year after TAVR are summarized in Table 3. These data were available at 1 year for 1,126 of the 1,363 living patients (82.6%). HD patients showed a significantly higher peak flow velocity and mean PG than non-HD patients. No significant differences were observed in other parameters between the HD and non-HD groups 1 year post-TAVR.

**Table 3:** One-Year Outcomes

	Prematched Cohort			Matched Cohort		
	HD	Non-HD	*P* Values	HD	Non-HD	*P* Values
Left ventricular ejection fraction, %	60.0 ± 10.8	63.9 ± 8.2	<0.001	60.6 ± 10.6	62.2 ± 8.9	0.456
Effective orifice area, cm^2^	1.75 ± 0.48	1.75 ± 0.46	<0.001	1.69 ± 0.47	1.76 ± 0.42	0.245
Index effective orifice area, cm^2^/m^2^	1.16 ± 0.32	1.20 ± 0.32	0.114	1.12 ± 0.32	1.16 ± 0.27	0.084
Peak flow velocity, m/s	2.2 ± 0.52	2.1 ± 0.49	0.359	2.2 ± 0.55	2.1 ± 0.46	0.005
Mean pressure gradient, mm Hg	10.4 ± 6.2	9.9 ± 5.0	0.251	11.2 ± 6.9	9.6 ± 4.7	0.005
Paravalvular leakage ≥ mild	75 (29.0)	510 (31.4)	0.433	61 (35.5)	57 (30.8)	0.350
Paravalvular leakage						
None	113 (43.6)	682 (42.0)	0.047	66 (38.4)	74 (40.0)	0.536
Trivial	72 (27.8)	442 (27.2)		46 (26.7)	55 (29.7)	
Mild	69 (26.6)	440 (27.1)		56 (32.6)	50 (27.0)	
Moderate	4 (1.5)	61 (3.8)		3 (1.7)	6 (3.2)	
Severe	1 (0.4)	0 (0)		1 (0.6)	0 (0)	
NYHA functional class III or IV	3 (1.2)	17 (1.1)	0.873	33 (19.8)	45 (25.3)	0.192

Values are mean ± SD, or counts with percentages. *P* values from Fisher's test (2 × 2 comparison), chi-square test (n × 2 comparisons), or Student's *t*-tests (continuous parameters).

Abbreviations as in Table 1.

### 1-year clinical outcomes

**Figure 3:**
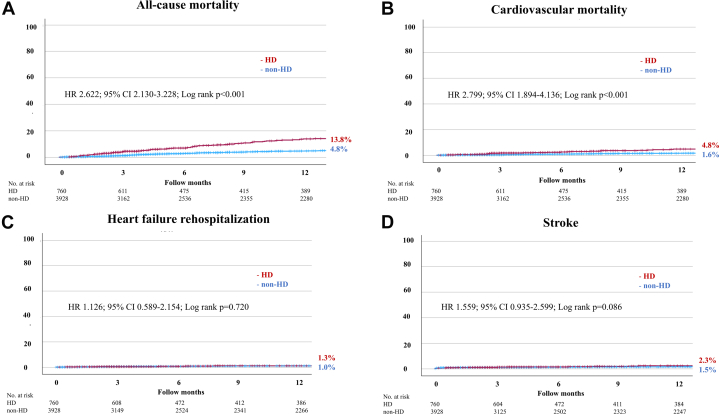
Kaplan-Meier Curves Depicting Clinical Outcomes for the Overall Cohort This figure shows time-to-event curves using the Kaplan-Meier method (censored at death or last valid contact in patients awaiting the subsequent follow-up or consent withdrawal), comparing overall patients with HD and non-HD. A Cox proportional hazards model was used to calculate HRs and 95% CIs for each clinical outcome. (A) All-cause mortality. (B) Cardiovascular mortality. (C) Heart failure rehospitalization. (D) Stroke. Abbreviation as in Figure 1.

**Figure 4:**
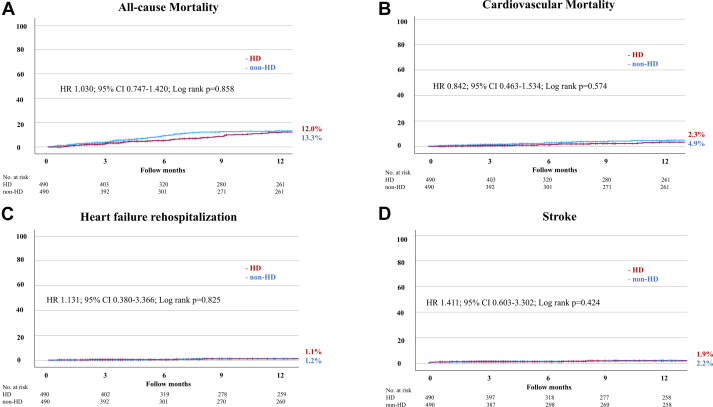
Kaplan-Meier Curves Depicting Clinical Outcomes for the Post-PSM Cohort This figure shows time-to-event curves using the Kaplan-Meier method (censored at death or last valid contact in patients awaiting the subsequent follow-up or consent withdrawal), comparing post-PSM patients with HD and non-HD. A Cox proportional hazards model was used to calculate HRs and 95% CIs for each clinical outcome. (A) All-cause mortality. (B) Cardiovascular mortality. (C) Heart failure rehospitalization. (D) Stroke. PSM = propensity score matching; other abbreviation as in Figure 1.

**Figure 5:**
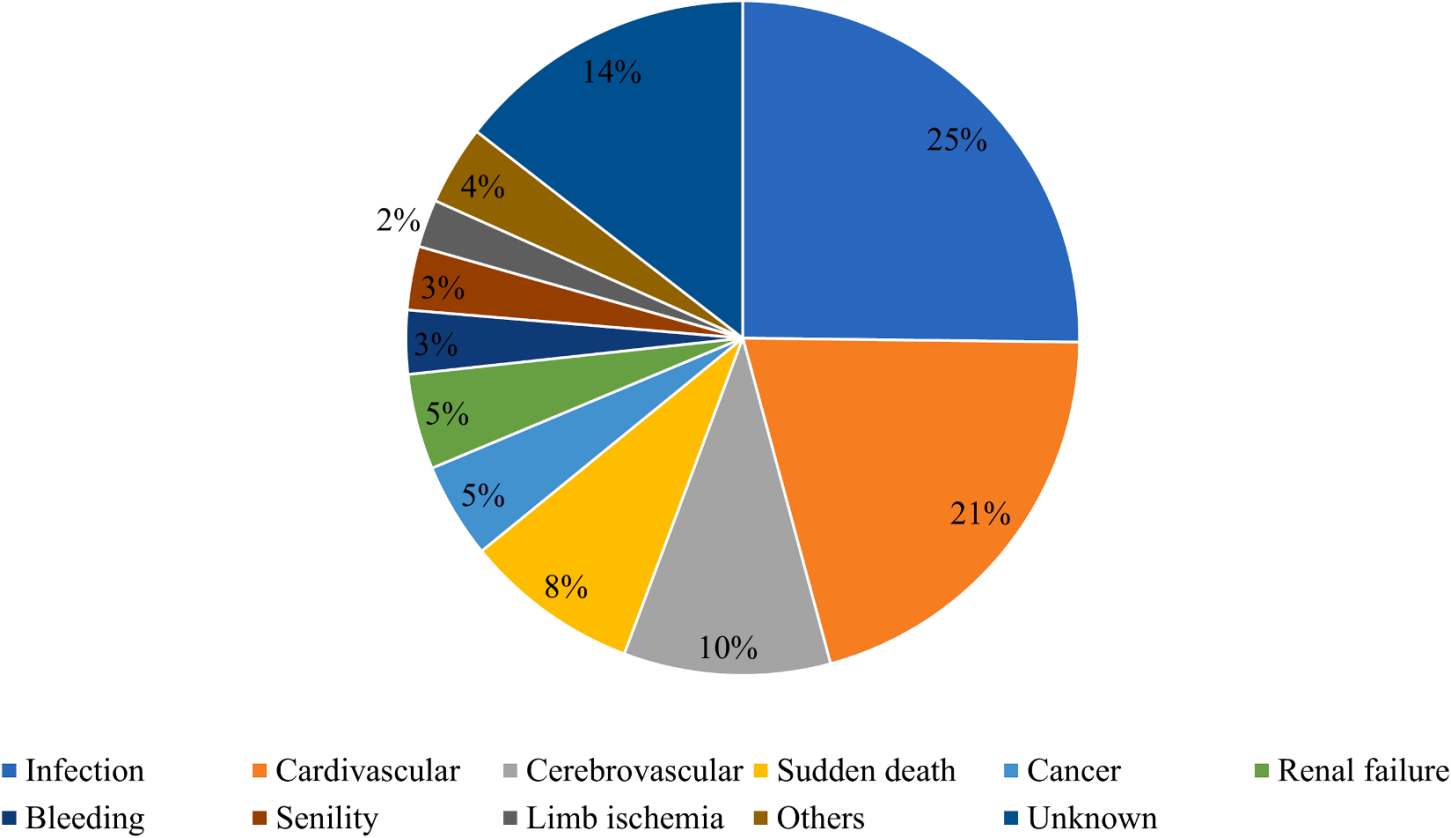
Cause of Death for Hemodialysis Patients This graph shows the distribution of causes of death among dialysis patients.

Among the HD patients in the PMS cohort, the follow-up duration for living patients was 208 [41-373] days. Kaplan-Meier curves depicting the clinical outcomes for both the overall and PSM cohorts, comparing HD and non-HD patients, are shown in Figures 3 and 4, respectively. Among the overall cohort, 1-year mortality occurred in 105 of 760 (13.8%) and 189 of 3,928 (4.8%)of patients in the HD and non-HD groups, respectively. The HD patients showed significantly higher all-cause and cardiovascular mortality than non-HD patients (all-cause mortality: HR: 2.622; 95% CI: 2.130-3.228; log-rank *P* < 0.001; cardiovascular mortality: HR: 2.799; 95% CI: 1.894-4.136; Log-rank *P* < 0.001). There were no significant differences in the incidences of HFH and stroke between the HD and non-HD groups (HFH: 10 of 760 [1.3%] vs 39 of 3,928 [1.0%], HR:: 1.126; 95% CI: 0.589-2.154; log-rank *P* = 0.720; stroke: 17 [2.3%] vs 59 of 3,928 [1.5%], HR: 1.559; 95% CI: 0.935-2.599; log-rank *P* = 0.086). In contrast, in the PSM cohort, no differences were observed in all-cause mortality (12.0% vs 13.3%, HR: 1.030; 95% CI: 0.747-1.420; *P* = 0.858), cardiovascular mortality (2.3% vs 4.9%, HR: 0.842; 95% CI: 0.463-1.534; *P* = 0.574), HFH (1.1% vs 1.2%, HR: 1.131; 95% CI: 0.380-3.366; *P* = 0.825), or stroke (1.9% vs 2.2%, HR: 1.411; 95% CI: 0.603-3.302; *P* = 0.424) between the 2 groups (Figure 4). The causes of death in HD patients who underwent TAVR in the overall cohort are summarized in Figure 5. The leading cause of death was infection (190 of 760 [25%]), followed by cardiovascular death (160 of 760 [21%]) and cerebrovascular death (76 of 760 [10%]).

**Figure 6:**
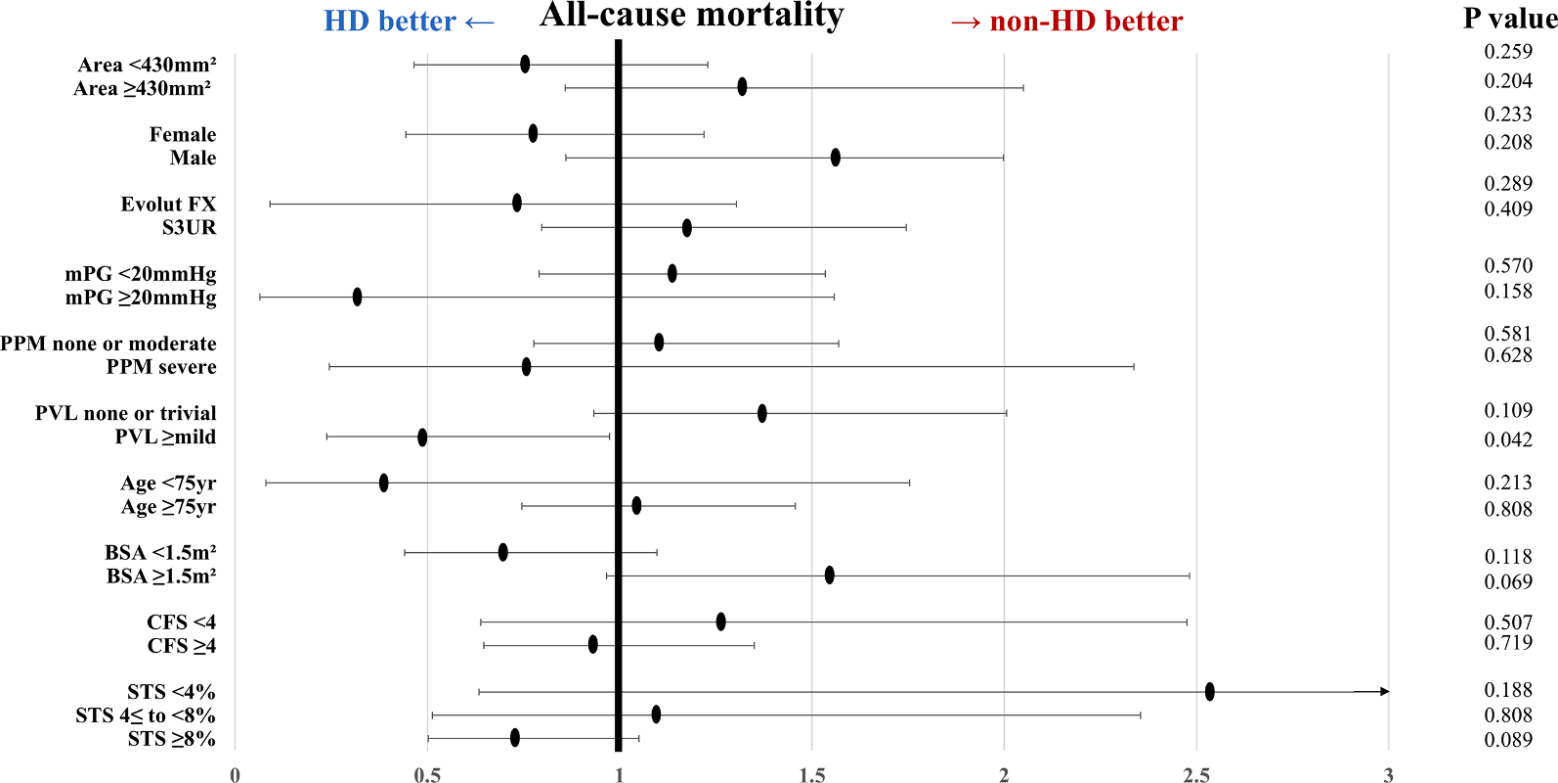
Comparison of 1-Year All-Cause Mortality Between HD and Non-HD Patients This figure shows subgroup analyses to assess the consistency of the treatment effect across clinically relevant subgroups. Treatment effects within each subgroup were estimated using Cox proportional hazards models, and HRs with 95% CIs were reported. BSA = body surface area; CFS = clinical frailty scale; mPG = mean pressure gradient; PPM = prosthesis-patient mismatch; PVL = paravalvular leakage; S3UR = SAPIEN 3 Ultra RESILIA; STS score = Society of Thoracic Surgeons score; other abbreviation as in Figure 1.

Subgroup analyses were performed using a forest plot to evaluate the differences in 1-year outcomes between HD and non-HD patients across potential risk modifiers for all-cause mortality, including annulus size (area and perimeter); sex; valve type; mean PG, PPM, and PVL; age; body surface area; CFS; and STS score. No significant interactions were observed for any of the variables except for a slight advantage in HD patients with mild PVL, as shown in Figure 6. We also analyzed all-cause mortality using multivariate analysis in a prematched cohort. In multivariable analysis, factors significantly associated with all-cause mortality included HD, male sex, body mass index, CFS ≥4, NYHA functional class III or IV, atrial fibrillation, prior stroke, STS score, hemoglobin level, severe PPM, and PVL ≥ mild (Table 4).

**Table 4:** Prematched All-Cause Mortality

	Multivariable Analysis		
	HR	95% CI	*P* Value
Hemodialysis	1.709	1.289-2.265	<0.001
Age	1.008	0.988-1.029	0.428
Male	1.449	1.089-1.927	0.011
Body mass index	0.934	0.903-0.966	<0.001
Clinical frailty scale ≥4	1.767	1.367-2.282	<0.001
NYHA functional class III or IV	1.849	1.466-2.333	<0.001
Diabetes mellitus	1.098	0.871-1.384	0.428
Atrial fibrillation	1.575	1.265-1.961	<0.001
Coronary artery disease	0.912	0.716-1.162	0.455
Peripheral artery disease	1.145	0.881-1.488	0.312
Previous stroke	1.567	1.197-2.052	0.001
Chronic obstructive pulmonary disease	1.054	0.710-1.567	0.793
STS score	1.034	1.023-1.045	<0.001
Hemoglobin	0.874	0.818-0.933	<0.001
Left ventricular ejection fraction	0.998	0.990-1.007	0.685
MDCT annulus area <430 mm^2^	0.833	0.635-1.092	0.185
Nonelective	0.921	0.647-1.311	0.648
Transfemoral	0.961	0.634-1.457	0.851
Self-expandable valves	0.787	0.614-1.009	0.059
Severe prosthesis-patient mismatch	1.466	1.008-2.132	0.045
Mean pressure gradient ≥20 mm Hg	0.735	0.271-1.998	0.546
Paravalvular leakage ≥mild	1.371	1.057-1.778	0.017

MDCT = multi-detected computed tomography; STS = Society of Thoracic Surgeons.

## Discussion

The salient findings of this study using the latest-generation devices are summarized in the Central Illustration and as follows. 1) HD patients had significantly more comorbidities and higher risk profiles than non-HD patients. After PSM, non-HD patients with a high-risk background were selected. 2) In the overall cohort, HD patients had higher mortality than non-HD patients at 30 days and 1 year, but these differences were attenuated following PSM. There were no significant differences in the secondary endpoints of cardiovascular death, HFH, and stroke between the 2 groups following PSM. 3) In multivariate analysis, HD remained an independent predictor of 1-year mortality even after adjustment, suggesting that renal dysfunction itself may still contribute to adverse prognosis beyond baseline comorbidities.

**Central Illustration:**
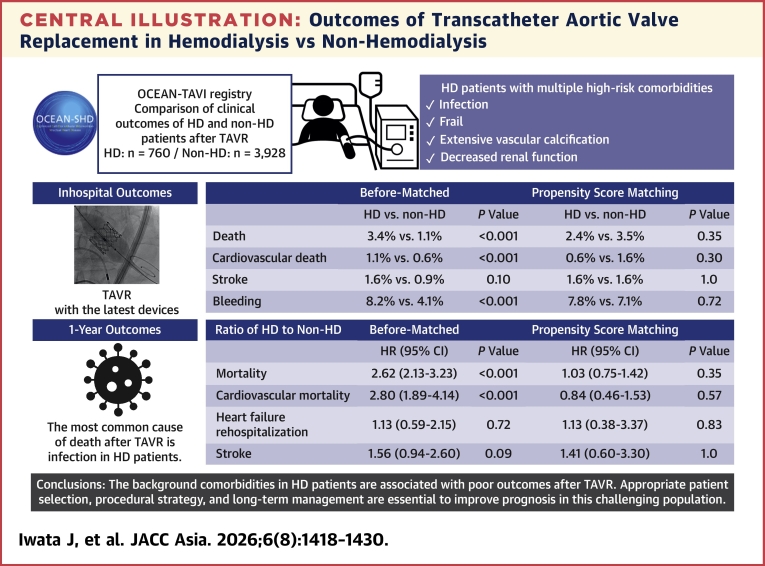
Outcomes of Transcatheter Aortic Valve Replacement in Hemodialysis vs Non-Hemodialysis This illustration summarizes results from the OCEAN-TAVI (Optimized transCathEter vAlvular iNtervention-Transcatheter Aortic Valve Implantation) registry comparing patients undergoing transcatheter aortic valve replacement (TAVR) with and without hemodialysis (HD). HD patients had more comorbidities and higher risks of mortality and bleeding before adjustment. After propensity matching, outcomes were comparable between groups. Infection was the leading cause of death in HD patients. These findings emphasize careful patient selection and tailored peri-procedural management in this high-risk population.

HD patients undergoing TAVR presented with substantially greater comorbidity burdens and higher operative risks than non-HD patients, reflecting their inherent frailty, low left ventricular ejection fraction, and frequent cardiovascular disease, consistent with previous findings.^9^, ^10^, ^11^^,^^13^ Following PSM, non-HD patients with similar high-risk backgrounds were selected to create 2 clinically comparable cohorts. This process inevitably led to “risk inflation” in the non-HD group, with patients with multiple comorbidities included to match the HD cohort.

In the short-term outcomes, HD patients had higher procedural risk, as reflected in STS scores before and after matching (11.6% vs 6.2% before; 10.4% vs 7.6% after). However, this risk model, originally designed for SAVR,^14^, ^15^, ^16^ will overestimate the procedural risk in HD patients undergoing TAVR. Consistent with this, in-hospital mortality was higher in HD patients before matching (3.4% vs 1.1%), although it became numerically lower after matching (2.4% vs 3.5%). This reversal suggests that, unlike SAVR, where HD markedly increases surgical mortality,^17^ HD itself does not inherently elevate the perioperative risk in TAVR. Instead, when non-HD patients with similar comorbidities were analyzed, their short-term outcomes deteriorated to comparable levels, indicating that procedural outcomes were primarily determined by baseline comorbidity burden rather than dialysis status.

At 1 year, the all-cause and cardiovascular mortality rates were nearly identical between the HD and non-HD patients in the matched cohort, indicating that adjustment for baseline comorbidities can largely attenuate the prognostic disadvantage of HD. Likewise, there were no significant differences in the secondary endpoints, including cardiovascular death, HFH, and stroke, supporting the interpretation that the worse outcomes observed before matching mainly reflect the cumulative burden of frailty and systemic comorbidities rather than HD itself. Notably, infection remains the leading cause of death among HD patients, consistent with national epidemiological data,^18^ suggesting that noncardiac factors play a dominant role in shaping long-term prognosis, even after technically successful TAVR.

In the overall cohort, HD patients demonstrated significantly higher mortality than non-HD patients, consistent with the results of previous short- and mid-term studies reports.^9^, ^10^, ^11^^,^^13^ Although recent reports using smaller registries have shown no differences in 1-year mortality rates, these findings may be limited by insufficient statistical power.^19^ In our PSM analysis, clinical outcomes, including primary and secondary endpoints, were comparable between HD and non-HD patients for up to 1 year after TAVR. This indicates that balancing the baseline comorbidities can significantly attenuate the prognostic gap between the 2 groups. However, HD remained an independent predictor of 1-year mortality in the multivariate analysis, suggesting that HD per se continues to contribute to adverse outcomes beyond the influence of comorbidities. This residual risk likely reflects the systemic consequences of end-stage renal disease, such as chronic inflammation, malnutrition, and infection susceptibility, which are not fully captured by baseline variables.^20^^,^^21^ Therefore, HD should be recognized not only as a marker of high comorbidity burden but also as an intrinsic risk condition that fundamentally limits long-term prognosis. From a clinical perspective, careful patient selection, optimized procedural planning, and aggressive management of modifiable factors are essential to minimize the risk and maximize the potential benefits of TAVR in this population.

### Study limitations

First, the study was based on an observational, unblinded, nonrandomized registry; therefore, residual confounding factors could not be completely excluded despite PSM. Second, echocardiographic assessments were performed at each participating institution according to the Valve Academic Research Consortium-3 criteria, which may have introduced interobserver variability. Third, the follow-up period was limited to 1 year. Given the chronic and progressive nature of end-stage renal disease, long-term outcomes should be evaluated in future studies. Fourth, this registry did not include Agatston scores. Consequently, it is difficult to compare the aortic valve calcification between HD patients and non-HD patients. Fifth, follow-up data regarding quality-of-life assessments were not available in our database. However, for the NYHA functional class at 1 year after TAVR, there was no significant difference between HD and non-HD patients, either before or after matching. This suggests that symptom improvement following TAVR is maintained equally well in HD patients as in non-HD patients. Finally, because the cohort primarily reflected Japanese clinical practice, the generalizability of the findings to younger or more active patients on HD may be limited. Nevertheless, the large sample size and high-quality echocardiographic follow-up of the registry enabled robust real-world comparisons between HD and non-HD patients undergoing TAVR.

## Conclusions

HD patients undergoing TAVR showed higher rates of the primary endpoint at 1 year than non-HD patients. However, in-hospital mortality was low, indicating that TAVR can be performed safely in HD patients, despite the high operative risk. After PSM, the differences in both the primary and secondary endpoints were largely attenuated. Nevertheless, HD remained an independent predictor of 1-year mortality, suggesting that end-stage renal disease itself imposes a residual systemic risk beyond baseline comorbidities. Careful patient selection, procedural optimization, and long-term management are essential for improving outcomes in this challenging population.

## Funding Support and Author Disclosures

The OCEAN-TAVI registry is supported by Edwards Lifesciences, Irvine, California, USA; Medtronic, Minneapolis, Minnesota, USA; Boston Scientific, Marlborough, Massachusetts, USA; Abbott, Chicago, Illinois, USA; and Daiichi-Sankyo, Chuo-ku, Tokyo, Japan. Drs Shimizu and Nishioka are clinical proctors at Edwards Lifesciences. Drs Tsuruta and Izumo are screening proctors at Edwards Lifesciences. Dr Ishizu is a proctor of intracardiac echocardiography at Johnson and Johnson. Drs Yashima and Nishina are clinical proctors at Medtronic. Drs Ohno and Shimura are clinical proctors at Medtronic and Abbott Medical. Drs Yamamoto, Shirai, Naganuma, Watanabe, Nakazawa, Ueno, Yamasaki, Asami, Hachinohe, Fuku, Tada, and Hayashida have served as clinical proctors at Edwards Lifesciences, Abbott Medical, and Medtronic. All other authors have reported that they have no relationships relevant to the contents of this paper to disclose.
